# Experiences and effects of telerehabilitation services for physiotherapy outpatients in a resource-constrained public health set-up in the backdrop of the COVID-19 pandemic: A proposal

**DOI:** 10.4102/sajp.v77i1.1528

**Published:** 2021-06-30

**Authors:** Humairaa Ebrahim, Prithi Pillay-Jayaraman, Yehudit Leibovitz, Nirvashi Naidoo, Tracey Bulmer, Bulelwa Bull, Sandy Lord, Monique M. Keller

**Affiliations:** 1 Department of Physiotherapy, Faculty of Health, Chris Hani Baragwanath Academic Hospital, Johannesburg, South Africa; 2 Department of Physiotherapy, School of Therapeutic Sciences, Faculty of Health Sciences, University of the Witwatersrand, Johannesburg, South Africa

**Keywords:** physiotherapy, telerehabilitation, orthopaedics, neuromusculoskeletal, paediatrics, COVID-19 pandemic

## Abstract

**Background:**

The announcement of a national lockdown in South Africa had country-wide impact on the delivery of health services. Strategies included prioritisation of patients and protecting patients who were considered at risk, resulting in the need for cancellation and temporary termination of many outpatient therapy services. This necessitated the urgent need to come up with a way of delivering physiotherapy rehabilitation services to patients in a more non-traditional format. Telerehabilitation allows for the provision of services by using electronic communication, thus ensuring that patients are still able to access necessary rehabilitation services.

**Methods/design:**

This is a prospective, mixed method study with participants recruited from the outpatient physiotherapy department of Chris Hani Baragwanath Academic Hospital (CHBAH). Telerehabilitation services will be provided via the patients’ preferred method of communication. On discharge, participants and therapists will be asked about their experiences of telerehabilitation.

**Discussion:**

Because of the current coronavirus disease 2019 (COVID-19) pandemic, patients are unable to receive traditional face-to-face physiotherapy services. Telerehabilitation offers a suitable alternative to treatment, but the feasibility, outcome and experiences of offering these services in the public health system have not been studied.

**Conclusion:**

This study will determine whether telerehabilitation is a feasible service that can be offered in the COVID-19 pandemic, as well as post-pandemic, to enable physiotherapists to access those patients who are often unable to attend physiotherapy because of transport costs and various other reasons for non-attendance.

**Clinical implications:**

The results of this study may indicate a way of managing patients in situations where face to face therapy cannot be undertaken.

**Protocol identification:**

Pan African Clinical Trial Registry, PACTR202103637993156.

## Introduction

On Thursday, 05 March 2020, the National Institute for Communicable Diseases confirmed the first positive case of coronavirus disease 2019 (COVID-19) in South Africa. This led to the announcement of a National State of Disaster by President Cyril Ramaphosa, and a 21-day lockdown was implemented on 26 March 2020. The National State of Disaster, as well as the 21-day lockdown, had tremendous implications for health professionals in terms of the manner in which services had to be prioritised and delivered. Shortly thereafter, the Department of Public Service and Administration issued Circular 18 of 2020 mandating healthcare institutions to find means to deliver healthcare services to patients by using online platforms rather than visiting physical service points. Gauteng Department of Health employees were encouraged to practise telehealth during the COVID-19 pandemic as a means of ensuring continuity of care whilst simultaneously reducing the risk of infection for patients, caregivers and healthcare professionals. Hence, it necessitated an urgent need to find ways to access the public (existing clients or patients) through the use of telephone and other online means.

Telerehabilitation refers to the mode of delivering rehabilitation services and public health awareness via information and communication technologies to facilitate the diagnosis, consultation, treatment, education, care management and self-management of a patient’s healthcare, whilst the patient is at the originating site and the healthcare provider is at the distant site (Chaet et al. 2017). Terminologies such as telerehabilitation and telephysiotherapy, which are branches of telehealth, are used interchangeably to describe the delivery of rehabilitation services to patients over the telephone or via an email or text message (Keerthi, Chandra & Deepak 2013). Telerehabilitation is thus delivered at a distance with the use of electronic communication and information technologies in the comfort of patients’ homes (Shaw 2009). This method of rehabilitation may be more feasible for the patient and hospital (Kuether et al. 2019) and will improve access for patients facing challenges because of transport and geographical considerations (Peretti et al. 2017). The success of a telerehabilitation session is dependent on the therapist linking the intervention content (cognitive behavioural therapy, education, reminders about self-management) and the type of telerehabilitation delivery (short message service [SMS] or cellular phone apps). Hence, this approach to service delivery provided the solution to the challenges posed by the COVID-19 pandemic.

Historically, telehealth implementation in South Africa was poor because of a lack of awareness and knowledge on the scope of practice (Mars 2011), as well as the Health Professions Council of South Africa (HPCSA) guidelines, which prevented the use of telehealth services by healthcare professionals (HPCSA 2014). Previously as well there had been usage of emails and telephone calls, but this was limited to general follow-up with patients or to serve as reminders for group classes. Whilst literature exists as to the benefits of telerehabilitation (Cason 2009; Kuether et al. 2019; Movahedazarhouligh et al. 2015; Odole et al. 2015; Peretti et al. 2017), there are limited studies that explore the use of telerehabilitation in public health settings in developing countries (Movahedazarhouligh et al. 2015). The potential benefit for telerehabilitation is greater in a developing country, especially in an under-resourced setting (Combi, Pozzani & Pozzi 2016).

As a result of the COVID-19 pandemic, the HPCSA and the South African Society of Physiotherapists (SASP) modified their stance and issued new guidelines on telerehabilitation services (HPCSA 2020; SASP 2020). Additionally, in the guidelines for telerehabilitation provided by the SASP, the use of telerehabilitation in many different fields of physiotherapy was explored, but not elaborated for use in orthopaedic conditions such as arthroplasty and chronic low back pain. Because of the lack of literature on telerehabilitation implementation in a public health context and to answer the challenges posed to the delivery of telerehabilitation services because of the COVID-19 pandemic, this proposal was created to streamline the mode of delivery of telerehabilitation. In addition, it aims to measure the effects of delivery of outpatient physiotherapy services by means of telerehabilitation as well as record the patient’s/caregiver’s and physiotherapist’s experiences.

### Problem statement

As a result of the COVID-19 pandemic, many patients who required outpatient rehabilitation services had not been able to attend the physiotherapy department to access the care they require to manage their conditions and prevent disability. As the pandemic progressed, the risk of contracting COVID-19 for those who attend public health facilities remained high, as the risk of exposure increased. The shift in the guidelines and legislations surrounding the practice of telerehabilitation as of March 2020 permitted its implementation for healthcare professionals in South Africa. Because of the existing gap in literature regarding telerehabilitation in public health context, this study will explore the effectiveness and experiences of telerehabilitation.

### Significance of the study

Because of the implications of social distancing and the responsibility of health professionals to assist in the promotion and protection of the health of the population, the use of online platforms to deliver healthcare services has become an urgent need. This study will help to determine the feasibility of using telerehabilitation to deliver services to orthopaedic, neuromusculoskeletal (NMS) and paediatric patient populations in the public health setting. In addition, this study will also assist in serving as operational research to investigate the long-term feasibility of a telerehabilitation approach in terms of staffing, resources, cost, and access for patients and in the long run serve to reduce the burden of disease in this population group.

### Objectives

The objectives of the study are as follows:

- To determine the patient and/or caregiver and physiotherapist experiences in the rollout of telerehabilitation for selected outpatient groups.
- To determine the effects of telerehabilitation services for patients who have orthopaedic conditions (arthroplasty), NMS conditions (chronic low back pain) and paediatric conditions (cerebral palsy, developmental delay and orthopaedic conditions).

## Methods and design

Purposive sampling will be used for this prospective mixed method study. Patients who have previously attended the outpatient physiotherapy department for the management of their orthopaedic, NMS and paediatric conditions prior to the national lockdown and those who had to have their appointments cancelled will be invited to participate. These patients will be selected as they are long-term patients requiring ongoing physiotherapy to prevent further disability but are not prioritised for face-to-face consultations because of the COVID-19 pandemic. Initially, a pilot study will be conducted to determine the sample size and rule out any errors in the methodology, and thereafter the study will be expanded.

A standard operating procedure (SOP) will be developed for arthroplasty (orthopaedic) and patients with chronic low back pain (NMS), cerebral palsy, developmental delay and acute orthopaedic conditions (paediatrics) with the purpose of providing an outline for therapists to deliver telerehabilitation services effectively and ethically. For each area, a database will be created containing videos and images of relevant interventions related to the specific condition being treated. Exercise prescription will commence from the patients’ current level of function and will be progressed as per the criteria described in the section’s guidelines of management of arthroplasty, chronic low back pain and paediatric patients. A section on general advice and health education will also be included.

Available methods of delivering telerehabilitation services include telephone calls, text messages (SMS or WhatsApp) and emails. Two modes of communication will be used for every participant, and this will be determined by the resources the patient has availed. The participant will initially be contacted via a telephone call, and the concept of telerehabilitation will be explained to them. Informed consent will be obtained from the patient for participation in the research, along with the consent for audio and/or video recordings of the session; therapists are to send or receive video and audio recordings as well as the caregiver to send or receive video and audio recordings should the patient not have a suitable phone. The preferred language of delivery (English or Zulu) will be obtained verbally from the participant prior to proceeding with telerehabilitation services. The mode of delivery of telerehabilitation services will be established at the initial contact. A suitable date and time for each session will be agreed upon between the physiotherapist and the participant. The type of delivery of information and the model of the patient’s phone will guide the extent of information transferred.

The participant and two physiotherapists will be present at each telerehabilitation consultation. Two physiotherapists will be present, as one physiotherapist will provide the telerehabilitation service and the other will record all necessary information, that is, the scribe. The call should therefore be on loudspeaker, in a private office to ensure confidentiality.

Permission will be obtained from the patient to record the session, and it will be an audio recording or video recording depending on how the session is conducted with the patient. The session will be recorded by using a specific app or device. This will be downloaded and stored on Google drive and will be backed up onto an external hard drive. The same will be done for the physiotherapy notes. All will be anonymised and there will be no names or identifying information on the recording.

Consistency and reliability during the proposed telehealth sessions will be ensured by following the telehealth guidelines set out by the SASP during 2020 and the HPCSA in 2014 and 2020. Participants will be asked to provide feedback for the orthopaedics and NMS subgroup and to complete a questionnaire for the paediatric population to provide their perceptions and experiences of telerehabilitation. For all interactions, attempts will be made to communicate with participants in their language of choice.

### Specific procedure for each of the conditions follows orthopaedic physiotherapy (arthroplasty)

#### Procedures

A database of exercises will be created with different categories and levels of exercises that include range of motion, strength and balance and are subdivided into easy, intermediate and difficult levels. Mobilisation videos (safety with walking frame, mobilising with two elbow crutches and mobilising with one elbow crutch) will also be included. Material on health education and various aspects of their condition and any co-morbidities that they have will also be compiled for dissemination at the appropriate time as determined by the therapist’s interaction with the participants.

At the initial session, a thorough assessment will be carried out by asking the sequence of questions shown in Table 1.

**TABLE 1:** Assessment questions.

Number	Question
1	How are you doing at home?
2	What is your pain now?
3	How are you mobilising at home?
4	What distance are you managing to mobilise at home?
5	How are your exercises going on at home?
6	What exercises are you doing at home?
7	How are you managing activities of daily living (ADL) such as eating, dressing and personal hygiene activities?
8	What are you struggling with at the moment?

*Source:* Ebrahim, H., Pillay-Jayaraman, P. & Leibovitz, Y., 2020, *Standard operating procedure for the use of telehealth in COVID-19 scenario by physiotherapists at Chris Hani Baragwanath Academic Hospital*, Chris Hani Baragwanath Academic Hospital, Johannesburg.

The framework questions shown in Table 1 will also be translated into Zulu, the language most spoken amongst the study population, and this will form the Arthroplasty Telerehabilitation Patient Notes (Appendix 1). Patients will be asked the questions in the language of choice (English or Zulu). If the patient’s language preference is anything other than English, then a therapist fluent in that language will be the primary person facilitating the telerehabilitation session. This further clarifies why each session will include two physiotherapists because at least one and when possible both physiotherapists will have to be fluent in the patient’s language. Alternatively, a family member will be asked to participate in the telerehabilitation session after getting informed consent, and they will be able to converse with the physiotherapist. The questions will be posed in the patient’s chosen language by the physiotherapist to the patient or family member. The responses will then be translated into English by the other physiotherapist for the purpose of record keeping. Member checking will be performed to validate the responses.

Based on the responses obtained from the patients, a home exercise programme (HEP) with the instructions on how many repetitions and how often to do the exercises will be sent to the patients via their preferred means of communication. To check the accuracy of the patients report on the consistency of performing the exercises, the information will be cross-checked with a family member on obtaining consent from the patient. In addition, the patients will be asked to send a video of them performing one of the exercises prescribed as chosen randomly by the therapist.

At subsequent telerehabilitation sessions (scheduled approximately 3–4 weeks apart), the therapist will obtain feedback on the patient’s functional status at home by using the subjective assessment framework. Follow-up sessions will consist of a re-assessment, patient education including pain management principles, medication adherence, importance of rehabilitation, ensuring that contraindications and precautions are understood and adhered to and scar management. Patient safety will also be addressed by giving suitable advice. Exercises will be prescribed, revised and progressed as required, and patients’ concerns will be addressed. Participant compliance will be monitored by encouraging the patient to keep a rehabilitation diary in which they record their exercises and progress as well as by corroborating the information with the family member. Weekly reminders will be sent, encouraging the patient to continue with their exercise programmes. Once patients have reached their functional goal, they can be discharged.

#### Outcome measures

The Hip Disability and Osteoarthritis Outcome Score Joint Replacement (HOOS JR), which is a self-reported questionnaire on performance pain and functional status, with good reliability and construct validity, will be used for patients with hip arthroplasty (Lyman et al. 2016). The Knee Injury and Osteoarthritis Outcome Score (KOOS JR) will be used for knee arthroplasty patients. The KOOS JR is a knee-specific outcome measure assessing pain, symptoms and function related to their knee and associated problems (Lyman et al. 2016). The KOOS has been validated in multiple populations, activity levels and ages. Divergent and convergent construct validities have been determined when comparing to the SF-36 a health survey. The HOOS JR and KOOS JR will be used as an objective outcome measure for participants with arthroplasty.

### Outpatient chronic low back pain physiotherapy specialist area

#### Procedures

The sample will consist of participants who had been assessed as being suitable for group exercise classes, after having completed individual face-to-face physiotherapy sessions. A similar procedure as for arthroplasty telerehabilitation session will be followed for the above cohort of patients. The telerehabilitation session will follow an adapted framework of questions, which will explore the patient’s functional ability, pain score and self-management principles (Appendix 2). At every session, the Keele STarT Back screening tool will be used as the outcome measure to monitor patient progress and to categorise patients as low, medium and high risk.

Following the first session of telerehabilitation, those patients who fall into the high-risk category will be called back for a face-to-face consultation session and managed accordingly. Patients who fall into the medium- and low-risk category will be managed via telerehabilitation, wherein a specific category or level of a HEP will be prescribed and sent to the patients via their preferred method of communication. Follow-up appointments will be at 2–4-week intervals. Based on the patients’ report and presentation, exercises and advice will be progressed. Once patients have reached a score of less than three on their Keele STarT Back screening assessment tool, they can be discharged from physiotherapy.

#### Outcome measure

The Keele STarT Back screening tool is available in English and Zulu, and according to Bruyère et al. (2014) the test-retest reliability of the Keele STarT back screening tool total score was excellent with an intraclass correlation coefficient of 0.90. The validity was found to be moderate to high in Pearson’s correlation for questions three to nine and a low correlation for questions one and two (Bier et al. 2017). This tool is designed to screen primary care patients with low back pain and serves as a prognostic indicator for initial decision-making. This tool allows clinicians to group low back pain patients into three categories of risk.

### Paediatric physiotherapy speciality area

#### Procedures

The population for paediatrics will comprise children with cerebral palsy, developmental delay and acute orthopaedic conditions. A resource survey (Appendix 3) will be used to determine suitable participants for conducting telerehabilitation services. The main telerehabilitation SOP will thereafter be followed. In addition, because minor children are being treated, informed consent and assent will be obtained from the child participant as well as the primary caregiver depending on the age of the child. The telerehabilitation consultation will include a thorough subjective clinical assessment in accordance with the Subjective Framework Questions (Appendix 4).

Based on the assessment obtained from the participant, a HEP will be selected for the patient from the database. The database will contain videos and images of relevant physiotherapy interventions related to the conditions within the paediatric unit, based on various ages and developmental levels. These resources will be accompanied by basic inputs from speech and language therapy (SLT) and occupational therapy (OT). A section on general advice and health education information will also be included. The participant or primary caregiver will then be sent the links or material via their preferred mode of communication. Based on the information received and the therapist’s clinical reasoning, additional information may be included, for example, verbal advice on the progression of a safe HEP, usage/repair/re-ordering of assistive devices/orthotics and on the need to consult specialist doctors, dieticians, social services, psychiatry and other rehabilitation professionals. Initial appointments will be scheduled every 2–3 weeks, and subsequent consultations will be scheduled every 5–6 weeks.

At each follow-up session, the following will be obtained: feedback from the caregiver on the patient’s functional status, the response of the child to the HEP and feedback/concerns from the caregiver, by using the Subjective Assessment Framework. Based on the caregiver’s report, the HEP and advice will be progressed accordingly and referrals made. At the final consultation as determined by the condition of the patient, caregivers will be requested to provide feedback on their perceptions and experiences of telerehabilitation (Appendix 5). If less than satisfactory outcome with telerehabilitation is received, face-to-face sessions will be considered as an alternative to telerehabilitation. Once patients have reached their functional goal, they can be discharged. Feedback from the therapist on the telerehabilitation experience will be collected as shown in Appendix 6.

### Documentation

The following processes will be followed to ensure participant safety, accurate documentation and consistency of procedures:

- The date, duration and time of the telerehabilitation session must be recorded as part of the patient’s notes.
- It must be clearly stated that assent and/or consent for telerehabilitation was obtained from the patient and caregiver or parent.
- The patient and/or caregiver must clearly express their understanding and confirm that he or she agrees that the practitioner or therapist may engage via a telerehabilitation consultation.
- That the patient and/or caregiver understand(s) that the consultation will be done via telephone/video/the Internet conferencing technology and that he or she agrees thereto.
- The purpose of the telerehabilitation consultation is to assess and treat his or her condition, subject to the information provided by the patient and/or caregiver.
- Ensure detailed clinical record keeping for the session – in Subjective, Objective, Assessment and Plan (SOAP) format document findings. All findings will be based on information provided by the patient and/or caregiver.
- If a standardised outcome measure was used, this should be included in the patient’s records.
- The consulting practitioners name, the scribe’s name and the location of the session must be documented at the end of the records (HPCSA 2014).

### Data management

Data will be collected by using data sheets. All data obtained will be safely kept electronically over a period of 6 years if not published, and 2 years if published. Data will be backed up onto Google Drive and a password-protected external hard drive.

The informed consent and questionnaire hard copies will also be stored securely in a locked drawer in the researcher’s code-protected office for a period of 6 years. After a period of 6 years, the hard copies with participants’ information will be deleted and destroyed by the researcher. In the case of minors, health records will be kept until the minor’s 21st birthday as required by national legislation.

### Statistical analysis

All identifiers will be removed. Descriptive statistics will be used to analyse the gathered data during the time of the study. Frequencies and percentages for categorical data and means and standard deviations or medians and percentiles for numerical data will be calculated. The data will be tabulated; trends will be presented in the form of graphs. A statistician assisted with the planning of data analysis. The statistical analysis for each objective will now be presented:

1. To determine the effects of telerehabilitation services for patients who have orthopaedics conditions (arthroplasty).

Categorical variables (symptoms, pain, function and daily living, function, sports and recreational activities, quality of life) will be summarised by using frequencies and percentages. Bivariate analysis between groups for total hip replacements and total knee replacements will be performed by using Chi squared test if 25% of the expected frequencies are above five or by using Fisher’s exact test if the assumption is not valid. Similarly, bivariate associations between gender, age and compliance with the outcomes (symptoms, pain, function and daily living, function, sports and recreational activities, quality of life) will be assessed by using Chi squared test or Fisher’s exact test:

2. To determine the effects of telerehabilitation services for patients who have NMS conditions (chronic low back pain).

Categorical variables (symptoms, pain, function and daily living, function, sports and recreational activities, quality of life) will be summarised by using frequencies and percentages. Whilst age is a continuous/numerical variable and the outcomes being categorical variables, with three groups, an analysis of variance (ANOVA) test is used if data are normally distributed or Kruskal Wallis test is used if they are not normally distributed:

3. To determine the effects of telerehabilitation services for patients who have paediatric conditions.

Categorical variables will be summarised by using frequencies and percentages:

4. To determine the patient or caregiver and physiotherapist experiences in the rollout of telerehabilitation services.

Frequencies and proportions will be used to summarise categorical variables. The two samples proportions test will be used to compare if there are any differences between the Yes or No responses. Comparisons between proportions with categorical variables will be performed by using the Chi squared test with the condition that the assumption is met:

5. Qualitative information will be gathered from two sources. The therapist’s experiences will be collected as positive, negative, challenges and other, and if further subthemes emerge from those categories, it will be described verbatim. In the subjective framework of questions, patients are asked a directed open-ended question on their experience of the call and the telerehabilitation sessions. The responses will also be categorised into themes that emerge.

### Validity and reliability

Reliability will be ensured by using consistent standardised verbal instructions. Reliability will be improved by doing the data collection in a standardised environment and by implementing standardised procedures. Validity of the patients’ responses will be confirmed by member checking.

### Ethical considerations

Ethical clearance will be obtained from the University of the Witwatersrand Human Research Ethics (Medical) Committee. Permission will be obtained from the management of Chris Hani Baragwanath Academic Hospital (CHBAH) and the head of the physiotherapy department where the study is to be conducted. Once ethical clearance is obtained, the study will commence and continue for a period of a year to ensure a sufficient sample for statistical analysis.

Participants will not receive any remuneration for participating in this study. This study is completely voluntary, and the patient may choose to withdraw at any point, without giving a reason and at no risk or penalty. Confidentiality will be maintained by keeping the participants’ names anonymous and by using identifiers on the outcome measures and data-recording sheets. In the event of identifying patient or caregiver ‘distress’ during telerehabilitation sessions, immediate referral will be made to the CHBAH’s Psychiatry Department, and this will be recorded in the patient’s file. Procedure for referral is outlined in the Distress Protocol (Appendix 7). All ethical guidelines will be adhered to as stipulated by HPCSA regulations regarding the provision of telerehabilitation.

The ethical approval was received from the Human Research Ethics Committee (Medical) University of the Witwatersrand M200772. The ethical approval was provided with the condition that ethics training certificates of all researchers are provided.

### Ethical considerations specifically relating to the paediatric participants

The patient’s caregiver is at liberty to ask questions and seek clarification of the procedures and telerehabilitation in general. The patient’s primary caregiver may at any time ask that the telerehabilitation consultation process be stopped, without future prejudice. An alternative way of safe and ethical follow-up would be considered so that treatment is continued as possible. If the proposed route of telerehabilitation delivery cannot be done because of device or connectivity limitations, the child should still be followed up in other appropriate and safe ways. No videos or images of the child should be sent to therapists on social media platforms.

A ‘form 22’ will be completed in the case of any suspected ‘abuse’, and appropriate and immediate referrals will be made to social services. A copy of this form will be attached to the telerehabilitation recording template. The consulting practitioner’s name as well as the scribe’s name (if applicable) and contact details and location will be recorded. There will be adherence to child protection principles (in the *Children’s Act* 38 of 2005) at all times. The HPCSA guidelines for Privacy and Confidentiality and Protection of Personal Information will be adhered to at all times.

Continuity of care should be facilitated, and practitioners are to ensure that telerehabilitation services meet the same standards of professional, legal, quality and ethical standards as services provided in person. The patient’s caregiver should acknowledge the risks of telerehabilitation consultation in respect of the technology used or assessment made by the practitioner or any physical injury sustained by the child whilst carrying out the HEP. Every effort will be made by the practitioner to mitigate these.

## Discussion

The COVID-19 pandemic demanded a vast change in the dynamics of healthcare delivery. Despite telerehabilitation growing as a mode of healthcare delivery in recent years, its uses in public health settings have been limited because of the lack of awareness and reservations (Odole et al. 2015). Our study will therefore enable us to explore the implementation and application of telerehabilitation in a public health setting to provide continuity of care in the COVID-19 pandemic as well as its long-term application. Eriksson, Lindström and Ekenberg (2011) highlights that the relationship formed between therapist and patient is vital during rehabilitation and stresses that this should not be compromised during telerehabilitation. Our study is therefore designed to ensure that patient engagement is maximised during the session as patient satisfaction with services is crucial to ensure improved compliance. Furthermore, the procedure of our study has been adjusted to accommodate for limited resources in our setting as in our context therapists do not have access to bulk SMS system, appointment systems, ‘apps’, bulk email system, encryption software, readily available databases for exercises or additional funding for this. Most of our patients do not own smart phones, have limited or no access to Wi-Fi or do not have data or the money for data. Hence, the procedure was designed primarily to have telephonic conversation with the patients where the therapist calls the patient to send and receive WhatsApp messages or in the rare circumstances to send and receive emails. Funding will be applied for from various institutions for the purchase of necessary equipment such as Wi-Fi routers and external hard drives as well as data and/or airtime for participants and researchers.

Outcomes of the study will allow us to explore the effectiveness of telerehabilitation improving access to care, quality of care, short- and long-term outcomes, patient and therapist satisfactions and the use of human resources. Results from our study will therefore explore both clinical outcomes as well as perceptions of telerehabilitation to gain an in-depth understanding of its efficacy, thereby determining its role in a public health setting.

Findings will be disseminated by means of publications in relevant accredited journals like the South African Health System Review to advise policymakers on the efficacy of such systems. It will be presented at various conferences and symposiums and, in addition, will be presented in several forums like Physiotherapy Forum, Rehabilitation Forum, CHBAH Research Day, Ekurhuleni Research Day and other symposiums to disseminate the information in the context it was studied on.

### Limitations

The participants’ understanding and insight into their condition and their performance will impact the accuracy of the responses. The therapist will also have to accurately rely on the patients presenting themselves as well.

## Appendix 3

### Resource survey of devices and data for tele-health paediatrics: English

**Table:**

**Patient name:**	
**Diagnosis:**	
**Age:**	**Consent:**
**Contact no 1:**	
**Contact no 2:**	
Would you be interested in doing tele-rehab?	
In what area do you live?	
Do you have a landline at home?	
Do you have access to a cell phone or any smartphone?	yes – my own yes – someone in my family no – don’t have a cell phone
Do you have internet access at home?	yes no
Do you have an email address?	Yes – my own Yes – someone in my family No
Is there someone at home who can help you with completing a telerehabilitation programme and the setup of your phone?	

## Appendix 4

### Subjective assessment framework questions paediatrics: English

**Table:**

CATEGORY	ITEM	QUESTION TO BE ASKED
**Patient progress** and **general concerns** regarding the child	Progress/changes since last therapy visit	HOW HAS YOUR CHILD CHANGED SINCE YOUR LAST VISIT? ANYTHING NEW HE/SHE HAS LEARNT TO DO?
	General concerns about the child	WHAT ARE YOU WORRIED ABOUT CONCERNING YOUR CHILD?
**Physical activities/functional abilities/highest functional level**	Caregiver concerns	ARE YOU WORRIED ABOUT ANYTHING WITH YOUR CHILD’S **BODY**? ARE YOU WORRIED ABOUT WHAT HIS BODY CAN DO?
	Physical/gross motor abilities	WHAT CAN YOUR CHILD **DO** WITH HIS BODY? eg rolling, sitting, crawling, standing, walking, using hands for play and feeding.
	Current HEP	WHAT **EXERCISES** ARE YOU DOING WITH YOUR CHILD?
	Frequency of HEP	HOW **OFTEN** ARE YOU DOING THE EXERCISES?
	Available equipment at home	WHAT **TOYS or EQUIPMENT** ARE YOU USING TO HELP YOU DO THIS?
	Who carries out the HEP	**WHO** DOES THE EXERCISES WITH YOUR CHILD?
	Difficulties with the HEP	WHAT IS **DIFFICULT** OR NOT WORKING WELL WITH THE EXERCISES?
	Compliance with referral/s	DID YOU ATTEND……… (relevant clinic or service)?
		(NB: check status of **hips** and need for **X-ray or Orthopedic** follow up)
**Specific Physical difficulties**: gait, balance, muscle strength, joint stiffness (FOR ORTHOPAEDIC CASES)	Concerns about the strength of body or certain joint/s	DO YOU SEE A **WEAKNESS** IN ANY PART OF YOUR CHILD’S BODY?
	Concerns about the ROM of certain joint/s	ARE THERE PARTS OF THE BODY THAT DON’T **MOVE AS WELL** AS THEY SHOULD? (Ask about the specific joint/s and the amount of movement in each direction).
	Concerns about walking/gait	ARE THERE PROBLEMS WITH YOUR CHILD’S **WALKING**? PLEASE EXPLAIN…. IS HE USING ANYTHING TO HELP HIM WALK?
	Concerns about balance	DOES YOUR CHILD HAVE POOR **BALANCE** or IS CLUMSY?
	Current HEP	WHAT **EXERCISES** ARE YOU DOING WITH YOUR CHILD?
	Frequency of HEP	HOW **OFTEN** ARE YOU DOING THE EXERCISES?
	Who carries out the HEP	**WHO** DOES THE EXERCISES WITH YOUR CHILD?
	Difficulties with the HEP	WHAT US **DIFFICULT** OR NOT WORKING WELL WITH THE EXERCISES?
	Compliance with referral/s	DID YOU ATTEND……… (relevant clinic or service)?
**Communication/auditory/visual abilities**	Caregiver concerns	ARE YOU WORRIED ABOUT ANYTHING WITH YOUR CHILD’S **TALKING, RESPONDING, SEEING or HEARING?**
	Current HEP	WHAT **ACTIVITIES** ARE YOU DOING TO HELP THESE?
	Available toys/equipment at home	WHAT **TOYS or EQUIPMENT** ARE YOU USING TO HELP YOU DO THIS?
	Compliance with referral/s	DID YOU ATTEND……… (relevant clinic or service)?
		(NB: check status of **hearing** and need for **Audiology** follow up)
		(NB: check status of **vision** and need for **St John’s** follow up)
**Feeding and growth**	Caregiver concerns	ARE YOU WORRIED ABOUT ANYTHING WITH YOUR CHILD’S **EATING AND DRINKING?**
	Growth curve in the Road To Health Card (RTHC)	HOW IS YOUR CHILD **GROWING** IF YOU LOOK AT THE RTHC? (help the mom to look at the curve or ask for a photo of the growth curve)
	Specific difficulties	IS THERE OFTEN **COUGHING OR VOMITING** WITH FEEDING? (Ask about amount and frequency). HOW LONG DOES IT TAKE TO FEED YOUR CHILD? (ask about solids and liquids)
	Current HEP	WHAT **ACTIVITIES** ARE YOU DOING TO HELP YOUR CHILD?
	Compliance with referral/s	DID YOU ATTEND……… (relevant clinic or service)?
**Use of orthotics, walking aids, seating devices, standing devices**	Nature of devices/orthotics	WHAT **ITEMS (splints)** or **EQUIPMENT** IS YOU CHILD USING: eg to sit, stand, keep his feet and legs straight? Any SPLINTS for HANDS?
		(**NB**: think about buggy/wheelchair, standing device, AFO’s, key-stones, hand splints: soft or hard)
	Frequency of usage	How **OFTEN** are you using these? In the **DAY** or at **NIGHT?**
	Difficulties encountered	Are they **FITTING** WELL? Do you have any **PROBLEMS** using these? Are they **BROKEN** or TOO **SMALL**?
	Compliance with referral/s	DID YOU ATTEND……… (relevant clinic or service)?
**General COPING**	Caregiver feedback	ARE YOU **COPING** WITH YOUR CHILD? ANY CONCERNS or OTHER PROBLEMS?
**GAS GOALS SET**	Set modified GAS GOAL that is measurable without having to see the child or send images of the child.	1) What do you want your child to be able to do in 3 months’ time? 2) Something that will be possible for him/her? 3) Think about the body, play and communication. (then wait for caregiver’s response) 4) How about this? (give one or two ideas for caregiver to think about) 5) Would you like to set this as a goal for us to work towards? 6) Then in 3 months we will see how far he/she has come and how to help him/her further. ***(NB: allow the caregiver time to think about this and confirm with you at the next call if he/she is unsure)***
**GAS GOALS MET**	Evaluate at 3 months	7) Do you remember the goal we set for your child? 8) Do you think he/she has achieved this? 9) What makes you say this? ***(NB: help the caregiver to see what part/s of the goal the child has achieved/partially achieved)***

## Appendix 5

### Caregiver questionnaire English

Please answer questions 1 to 10 and give a brief comment and suggestion for all questions.

**Table:**

1.	Did you enjoy the experience of tele-rehab for you and your child?	Yes	No	N/A	Comments and/or Suggestions		
2.	Do you feel like you learnt better how to help your child during tele-rehab?	Yes	No	N/A	Comments and/or Suggestions		
3.	What was difficult for you during the tele-rehab calls and treatment?	Yes	No	N/A	Comments and/or Suggestions		
4.	Was your culture and language respected during the calls and treatment?	Yes	No	N/A	Comments and/or Suggestions		
5.	Was the therapist’s attitude friendly and caring towards you?	Yes	No	N/A	Comments and/or Suggestions		
6.	Were you able to use any of the videos or pictures that were sent to you?	Yes	No	N/A	Comments and/or Suggestions		
7.	Were you given the chance to ask questions about your child’s treatment?	Yes	No	N/A	Comments and/or Suggestions		
8.	Where you given the right to refuse tele-rehab treatment?	Yes	No	N/A	Comments and/or Suggestions		
9.	Do you feel your child has benefitted from the advice and exercises given during tele-rehab?	Yes	No	N/A	Comments and/or Suggestions		
10.	How likely are you to recommend tele-rehab to another parent?	Very low	Low	Not sure	High	Very high	Comment and/or suggestions:

## Appendix 6

### Feedback from therapists on their tele-health experience

Please score the below questions as per the scale shown and please give a brief comment and a suggestion/reason for all questions.

**Table:**

**1: Very Poor**	**2: Poor**	**3: Average**	**4: Good**	**5: Very Good**

**Table:**

Questions	1	2	3	4	5	Comments and/or Suggestions
1. Rate the effectiveness of the implementation of the tele-rehab						
2. Do you strive towards achieving the best physiotherapy practice while conducting tele-rehab?	Yes/No (Elaborate Please)					
3. How likely are you to recommend tele-rehab to other colleagues?	1	2	3	4	5	Comments and/or Suggestions
4. Name the two resources that will assist you in performing tele-rehab more efficiently.						
5. Do you feel that you are effective as a therapist during tele-rehab consultations?	1	2	3	4	5	Comments and/or Suggestions
6. Are you satisfied with your experience of tele-rehab overall?	1	2	3	4	5	Comments and/or Suggestions
7. Which skills (clinical and non-clinical) did you learn or develop to carry out tele- rehab more effectively?	Clinical: Non-clinical:					
8. Which of your skills (clinical and non-clinical) did you transfer (teach other staff) to aid in carrying out effective tele- rehab?	Clinical: Non-clinical:					
9. What protocols, procedures or logistics would help to provide more effective tele-rehab in future?						
10. Any other suggestions or comments?						
